# Evaluation of Cancer Cell Invasion Ability Based on Electrochemical Impedance

**DOI:** 10.3390/bios15020091

**Published:** 2025-02-06

**Authors:** Feiyang Jiang, Mingji Wei, Si Chen, Yanfei Wang, Ning Liu, Ning Yang

**Affiliations:** 1 School of Electrical Information Engineering, Jiangsu University, Zhenjiang 212013, China; 2 Fluid Machinery Center, Jiangsu University, Zhenjiang 212013, China

**Keywords:** cancer cell invasion, microfluidic, electrical impedance model

## Abstract

Cancer metastasis is the leading cause of cancer-related deaths, with the ability of cancer cells to invade blood vessels or lymphatic systems, determining their metastatic potential. Therefore, the rapid and accurate assessment of cell invasiveness is crucial. Current methods, such as the Transwell assay and fluorescent labeling, are complex, invasive, and may disrupt the physiological state of live cells. In this study, we introduce an electrochemical impedance-based method for evaluating cancer cell invasiveness, combining Transwell and microfluidic technologies to monitor the invasion process in a dynamic environment. A stable microfluidic chip with 30 μm interdigital electrodes was developed, optimized for HeLa cell detection. We identified 1 kHz as the optimal frequency for achieving the maximum impedance resolution of cancer cell invasiveness. By correlating the impedance response of Z_cells_/Z_no-cells_ with invasiveness, we established a reliable electrochemical model. This model was validated with a hydrogen peroxide cytotoxicity assay, showing a high correlation with optical staining and a minimal error of 1.89%, underscoring its potential for drug efficacy prediction. The proposed method offers rapid detection, low cost, and requires no manual intervention, making it an efficient and reliable tool for assessing cancer cell invasiveness in therapeutic research.

## 1. Introduction

Cancer metastasis is a leading cause of mortality in cancer patients, responsible for approximately 90% of cancer-related deaths [1]. The process of metastasis is complex and involves several stages, with the ability of cancer cells to detach from the primary tumor and enter the bloodstream or lymphatic system—referred to as invasion ability—playing a critical role in determining whether they can successfully form distant metastases [2,3]. Therefore, rapid and accurate assessment of cancer cell aggressiveness is crucial for predicting cancer progression, guiding treatment decisions, and informing the development of novel therapeutic strategies.

Most existing in vivo detection methods rely on mouse models [4,5]. While these models enable direct observation of cancer cell behavior in vivo, they are associated with long experimental durations, high costs, and limited suitability for reproducibility studies. However, most traditional in vitro cancer cell invasion models rely on the Transwell invasion assay [6,7]. The method involves using a chamber with a bilayer membrane structure where the upper membrane is coated with a matrix gel (e.g., Matrigel) that mimics the extracellular matrix. Cancer cells are inoculated onto the upper layer, and after a defined incubation period, they secrete matrix metalloproteinases (MMPs) to degrade the matrix gel and migrate through the membrane pores. Invasion ability is then assessed by counting the cells in the lower chamber. Liu et al. [8] developed a hexagonal Transwell chamber to assess tumor metastasis horizontally and conduct preliminary drug screening. Due to the need for complex manual operations, such as fixation, staining, and cell counting, along with the potential for cell damage, this method is terminal and does not allow for continuous monitoring. Moreover, its static environment fails to fully replicate the complex three-dimensional in vivo microenvironment.

Microfluidic technology offers a dynamic observation method, distinct from static detection techniques. Truong et al. [9] developed a 3D organoid microfluidic model to simulate the interaction between tumor and endothelial cells, studying the migration and invasion behavior of tumor cells within 3D hydrogels using immunofluorescence staining and high-resolution imaging. Wong et al. [10] studied the interaction between human invasive breast cancer cells and functional microvessels by constructing a tissue-engineered tumor-microvessel platform and employing live-cell fluorescence microscopy. Nagaraju et al. [11] developed a microfluidic tumor-vascular model to observe and quantify the invasion and endoscopy of breast cancer cells in a 3D extracellular matrix using fluorescence and phase contrast imaging techniques. They also evaluated bidirectional signaling in the tumor-vascular microenvironment. The combination of microfluidic technology with fluorescence or bioluminescence labeling enables the real-time, dynamic observation of cancer cell migration and invasion within a more biologically relevant environment, providing intuitive imaging evidence. However, fluorescence labeling is an invasive detection method that can damage living cells and disrupt their normal physiological state. Additionally, cell overlap can occur, which compromises the accuracy of optical imaging.

The electrochemical impedance method is considered a non-destructive, real-time, and quantitative automated detection tool [12], widely used in cell-related detection and various other fields [13,14,15]. Chitale et al. [16] designed a 96-well microplate platform based on electrical impedance technology, utilizing high- and low-frequency electric fields to reflect distinct cell characteristics and enabling real-time, label-free monitoring of cell function and morphology. Yang et al. [17] proposed an electrocellular matrix impedance sensing technology with ambient temperature control, used to simultaneously assess cell viability and survival rates. It is easy to operate, requires minimal time, is less dependent on sample conditions, and offers higher efficiency and accuracy, making it suitable for drug screening and cell physiological activity research. Additionally, the impedance method offers the advantage of easy integration. Wang et al. [18] combined electrical impedance with microfluidics to propose a tumor sphere diffusion and real-time impedance monitoring platform, which effectively monitors tumor sphere growth and their response to anticancer drugs. Therefore, the combination of microfluidic technology and impedance methods not only avoids the drawbacks of traditional staining methods but also allows for real-time monitoring under flow conditions.

In summary, we propose a novel method that integrates microfluidics, electrochemical impedance spectroscopy (EIS), and Transwell assays to evaluate cancer cell invasion capacity. This integration combines the spatial precision of microfluidics, the real-time monitoring capabilities of EIS, and the well-established Transwell assay into a single platform for comprehensive cancer analysis. Unlike traditional methods, our approach allows for non-invasive monitoring of cancer cell invasion without complex operations, cell labeling, or damage. A key innovation in our method is the development of an electrochemical impedance model under microfluidics, which efficiently quantifies cancer cell invasion capacity in a way that traditional assays cannot. This model is validated by comparison with the gold standard method, demonstrating its accuracy and reliability. Additionally, the platform’s ability to capture real-time electrical responses from cells during invasion offers new insights into the invasive behavior of cancer cells that were previously difficult to measure. By integrating these technologies, we provide a novel approach for evaluating cytotoxicity, offering a powerful tool for cancer drug screening. This method not only advances cancer research but also holds potential in early cancer detection and therapeutic monitoring.

## 2. Materials and Methods

### 2.1. Cell Culture

HeLa cells (Xinyu Biotechnology, Shanghai, China) were selected as the target cancer cell line. The reagents required for cell culture include DMEM (Shanghai Xinyu Biotechnology), fetal bovine serum (FBS) (Gibco, Carlsbad, CA, USA), 1% penicillin/streptomycin solution (P/S) (Gibco), and 0.25% trypsin-ethylenediaminetetraacetic acid (EDTA) (Life Technologies, Carlsbad, CA, USA). Following inoculation, the cells were placed in an incubator (Heal Force^®^, HongKong, China) set to a temperature of 37 °C and a CO_2_ concentration of 5%, allowing the cells to acclimate to the new environment and begin attachment. When the cells reach a density of 80–90% confluence, they are detached from the surface using trypsin, counted, and proportionally divided into new culture vessels for continued culture.

### 2.2. System Construction

**Figure 1:**
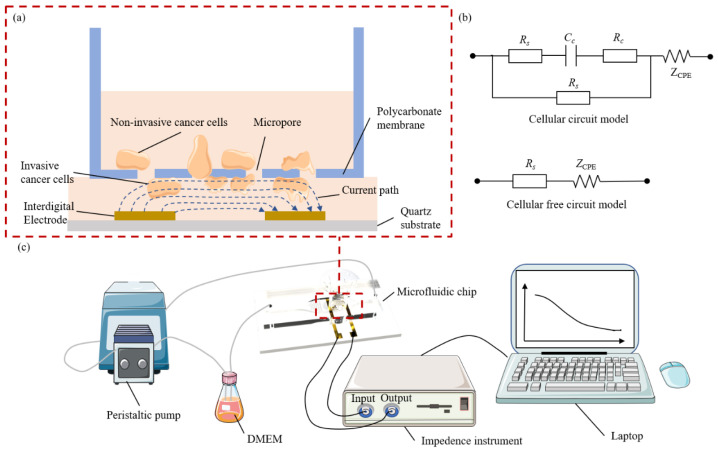
(**a**) Principle and current path of electrical impedance detection of cancer cell invasion ability. (**b**) Equivalent model of electrochemical measurement. (**c**) Design of cancer cell invasion ability evaluation system based on electrochemical impedance.

The electrical connection for the experiment utilizes a titanium interdigitated electrode, with quartz serving as the substrate material. We chose titanium electrodes because of their low cost, excellent corrosion resistance, and biocompatibility, while the finger-like electrodes offer higher electrochemical sensitivity due to their larger effective surface area and electric field distribution advantages, which are particularly suitable for microfluidic systems. The chip structure was designed using AutoCAD (2022). The fluid channel in the chip was designed as a long, strip-shaped structure, with a bent configuration at the entrance to ensure the complete mixing of the injected liquid. A microcolumn array was incorporated into the center of the channel to ensure a uniform flow rate in the detection area. A chip structure film was printed. An SU-8 adhesive was uniformly applied to the silicon wafer using a homogenizing machine (Sedekase Setas Electronics, Beijing, China), followed by film attachment and exposure to a curing channel with 365 nm ultraviolet light, and subsequent cleaning with the developer to form the chip mold. Polydimethylsiloxane (PDMS), known for its biocompatibility, was used for film placement. The heat-cured PDMS chip was treated with plasma using an ion bonding machine (PTL), bonded to a quartz substrate, and used to fabricate a microfluidic chip with a Transwell chamber (Corning, NY, USA). The overall chip was 76 mm long and 52 mm wide, and the PDMS part was 70 mm long and 17 mm wide. The channel was 2 mm at its narrowest point, 9 mm at its widest point, and 100 μm in height. Interfinger electrodes were 20 mm long and 10 mm wide. Figure 1c illustrates the system connection diagram. The resulting microfluidic chip was connected to an intermediate frequency impedance analyzer (MFIA) (Zurich Instruments, Zurich, Switzerland) for data acquisition and analysis, utilizing LabOne 24.10 software (Zurich Instruments, Zurich, Switzerland). The microfluidic channel was connected to a peristaltic pump through which the medium required for cancer cell culture flows, creating a dynamic invasion environment.

### 2.3. Transwell Method

The porous membrane of the Transwell chamber was pre-coated with a matrix gel, such as Matrigel, to simulate the extracellular matrix. The coated chamber was incubated at 37 °C for 3 h to solidify the matrix gel. The cells to be studied were digested into a single-cell suspension and adjusted to the appropriate concentration. The cells were counted to ensure an equal number across each experimental set. The cell suspension was added to the upper chamber of the Transwell, ensuring an equal number of cells in each well. The lower chamber was supplemented with a medium containing chemotactic factors (such as serum) to induce cell invasion toward the bottom. The Transwell chamber was placed in a cell incubator at 37 °C and 5% CO₂ and incubated for a specified duration (typically 24–48 h). After incubation, the cells were fixed and stained for analysis. The cells that passed through the membrane were observed and counted using a light microscope. A higher number of cells passing through the membrane indicates greater invasive capacity. Thus, cancer cell invasion was quantified by measuring the number of invasive cells per unit area (mm^2^). The total area of cell invasion was 33 mm^2^ of the bottom membrane area of the Transwell chamber, and the total number of cell invasion measured could also be calculated.

### 2.4. Principle of Simulation Analysis

We used COMSOL Multiphysics 6.0 to simulate the temperature, concentration diffusion, and flow field of microfluidic chips, and conducted channel optimization comparisons. The heat convection equation is as follows:

(1) $$\begin{array}{c} \rho C_{p}\cdot \frac{\partial T}{\partial t}+\rho C_{p}u\cdot \nabla T-k\nabla \cdot \nabla T=0 \end{array}$$

where ρ is the fluid density, T is the temperature at any point in the channel, C*_p_* constant pressure heat capacity, and k is the thermal conductivity of the fluid.

Concentration diffusion can be expressed by Fick’s law:

(2) $$\begin{array}{c} \frac{\partial c_{i}}{\partial t}-D_{i}\nabla \cdot \nabla c_{i}+u\cdot \nabla c_{i}=R_{i} \end{array}$$

where c*_i_* and D*_i_* are the concentration and diffusion coefficient in the medium in which component *i* is located, *u* is the velocity field of the fluid, and R*_i_* is the source term of component *i*.

The flow field *u* in the microfluidic chip is given by the Navier–Stokes equation combined with the continuity equation:

(3) $$\begin{array}{c} \begin{array}{c} \left(u\cdot \nabla \right)u=-\frac{1}{\rho }\nabla P+u\nabla^{2}u+f\\ \nabla \cdot \left(\rho u\right)=0 \end{array} \end{array}$$

where ρ and *P* are the density and pressure in the volume element, respectively, and *f* is the external force term.

### 2.5. Determination of Cell Sensitive Frequencies and Optimization of Electrical Signals

The detection voltage of the impedance meter was set to 30 mV. Before the experiment, DMEM complete culture medium was passed into the prepared microfluidic chip channel, and the cell-free impedance spectrum Z_no-cell_ was recorded at this time. Cancer cells were added to the invasion chamber, and after invasion for about 24 h, the frequency range of 100 Hz to 1000 kHz was scanned to record the cell impedance spectrum Z_cell-electronic_ at this time. We chose a higher frequency range (100–10 kHz) rather than 0.1 to 100 Hz because it provides higher signal-to-noise ratio and sensitivity, avoids noise interference caused by low frequencies, and more effectively captures dynamic changes in the electrochemical properties of cells. The invasion cell impedance spectrum Z_cells_ can be calculated as follows:

(4) $$\left|Z_{\mathrm{cells}}\right|=\left|Z_{\mathrm{cell}-\mathrm{electronic}}\right|-\left|Z_{\mathrm{no}-\mathrm{cell}}\right|$$

Z_cells/_Z_no-cell_ is the ratio of cellular impedance to cell-free impedance and represents the resolution of impedance [19]. When Z_cells/_Z_no-cell_ reaches the maximum and the fluctuation is small, the frequency currently *f*_0_ is selected as the cell sensitive frequency. The frequency of cell sensitivity depends on sample properties and electrode properties, including cell, medium, electrode conductivity, and detection voltage [20,21]. For specific samples and electrodes, selecting the appropriate cell sensitive frequency can obtain sensitive and stable results. Because of electrode polarization and electrical noise, inappropriate frequency will lead to inaccurate results. Usually, the sensitive frequency calculated from the first measurement is chosen as the measurement frequency of this experiment.

To explore the effect of electrode topology on our established electrochemical model, we need to screen different specifications of interdigital electrodes to achieve maximum cell resolution accuracy. The same invasion chamber is placed on different electrodes, and Z_cells/_Z_no-cell_ is measured and calculated. According to the principle of maximum resolution and stability, the appropriate electrode is selected to achieve the optimal measurement signal.

### 2.6. Equivalent Model of Electrochemical Measurement

In this paper, we establish an equivalent model of the electrochemical measurement of cancer cell invasion. Figure 1a shows the detection of the invasion electrical impedance of cancer cells and the schematic diagram of the current path. Cancer cells invade the micropore membrane and adhere to the bottom of the cell, which will change the current path of the interdigital electrode and present impedance information. Cells are electrically conductive, with the cell membrane regarded as a capacitor and the cytoplasm as a resistor [22,23]. Figure 1b shows the electrochemical equivalent circuit model of cell-free detection and cell-to-electrode suspension detection. The electrode-electrolyte surface is usually equivalent to a constant phase angle element CPE [24,25], which is calculated as follows:

(5) $$Z_{CPE}=K{\left(i\omega \right)}^{-n}$$

where K is the impedance amplitude of CPE, determined by the conductivity of the electrolyte, *i* is the imaginary unit ($i=\sqrt{-1}$), *ω* is the angular frequency of the AC signal (*ω* = 2π*f*, *f* is the AC frequency), and n is a constant between 0 and 1. If n approaches 1, the interface exhibits more capacitive features. On the other hand, if n approaches 0, the interface exhibits more resistive characteristics. The medium electrolyte is usually equivalent to pure resistance R_s_, while the cell membrane and cytoplasm are equivalent to capacitance C_c_ and resistance R_c_ in series. The sensing mechanism is that the current generated by the interdigital electrode has two paths: (1) electrolyte; (2) cell membrane. Then, the impedances of the cell-free and cell-electrode equivalent circuit models are as follows:

(6) $$Z_{no-cell}=R_{s}+K{\left(i\omega \right)}^{-n}$$

(7) $$Z_{cell-electronic}=\frac{R_{s}\left(R_{s}+C_{c}+R_{c}\right)}{2R_{s}+C_{c}+R_{c}}+K{\left(i\omega \right)}^{-n}$$

We examined the changes in invasion ability of Hela cells at the flow rate of 0, 1, 2, 3, and 4 mL/min for 12 h. The appropriate flow rate was selected, the invasion lasted for 24 h, the impedance data were recorded every 6 h, and the appropriate experimental conditions were selected for subsequent verification. To verify the validity of the model, we used it to monitor cytotoxicity experiments. We added different concentrations of hydrogen peroxide to the cancer cell invasion chamber to evaluate its effect on cancer cell invasion and compared the results of resistance and optical staining with conventional Transwell assay. In this paper, we performed experiments using three independent microfluidic chips, and at least three independent measurements were performed on each chip.

## 3. Results and Discussion

### 3.1. Chip Simulation

**Figure 2:**
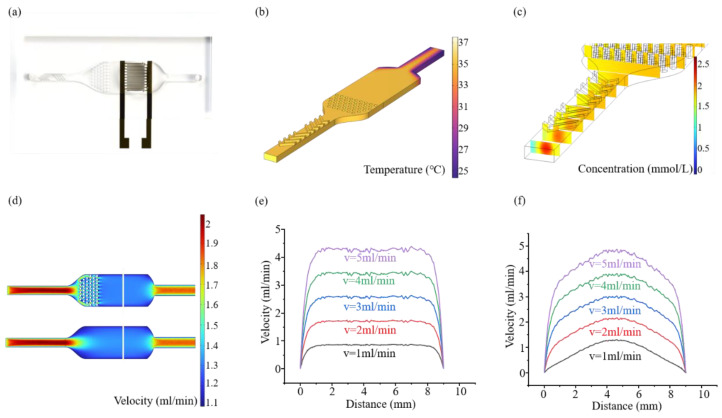
(**a**) Microfluidic chip structure design diagram. (**b**) Microfluidic chip heat loss simulation diagram. (**c**) Optimization simulation effect of bending structure at channel entrance for liquid mixing. (**d**) Simulation effect of liquid flow rate control with or without microcolumn array in the middle of channel. (**e**) Flow rate change in the detection area when there is microcolumn array. (**f**) Velocity change in the detection area without microcolumn array.

In order to better detect the invasion ability of Hela cells, it is necessary to continuously optimize the design of microfluidic chips. Figure 2a and Figure A5 show the schematic diagram of the microfluidic chip designed by us. Two groups of bending structures are designed at the entrance of the channel, the main function is to mix the solution and make the fluid concentration more uniform so as not to affect the subsequent impedance measurement. In the middle of the channel is a microcolumn array, which is used to keep the flow rate in the detection area stable. In order to maintain the invasion environment temperature of Hela cells, we passed a 37 °C culture medium, and the external temperature was 28 °C. Figure 2b simulates the overall heat loss of the chip, and the temperature of the detection area can be kept above 35 °C, which is suitable for the growth of Hela cells and close to the human body temperature. In Figure 2c, we simulated the mixing effect of the bending structure. The bending structure has a good mixing effect from the obvious concentration difference in the fluid cross section at the entrance to almost no concentration difference in the fluid cross section after passing the bending structure. Figure 2d is the simulation effect diagram of liquid flow rate control with or without micro-column array in the middle of the channel, and the white area in the figure is the selection point of the detection area in Figure 2e,f. We compared the changes in flow velocity in the detection area with or without microcolumn array. Although the center flow velocity is closer to the input flow velocity without microcolumn array, the flow velocity from the center to both ends changes greatly and is not as stable as that with microcolumn array. However, the invasion of cancer cells is affected by the flow rate of the fluid, and the unstable flow rate will cause the invasion of cancer cells to be uneven, thus affecting the final detection effect. Therefore, the design of the microcolumn array can maintain a stable flow rate, so that the cancer cells are evenly invaded, so as to achieve the best detection results.

### 3.2. Optimization of Electrical Signal

**Figure 3:**
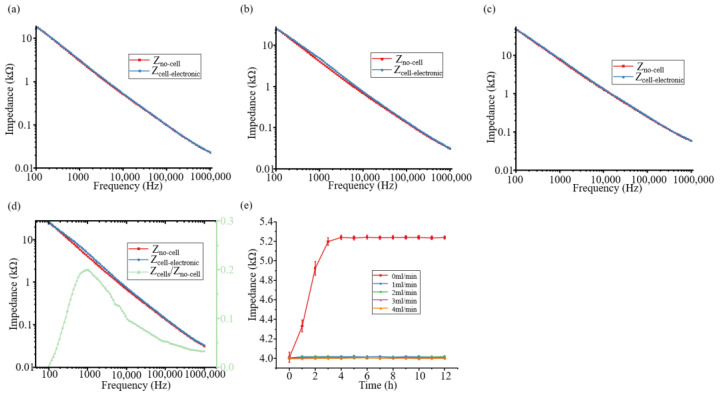
(**a**) Cell-free and cell-electrode impedance spectra from 100 Hz to 1000 kHz for interdigital electrodes with 5 μm finger spacing. (**b**) Cell-free and cell-electrode impedance spectra from 100 Hz to 1000 kHz for interdigital electrodes with 30 μm finger spacing. (**c**) Cell-free and cell-electrode impedance spectra from 100 Hz to 1000 kHz for interdigital electrodes with 50 μm finger spacing. (**d**) Relationship between Z_cells_/Z_no-cell_ and frequency. (**e**) Effect of different flow rates on cell-free electrode impedance.

Because the topology of the interdigital electrode will affect the resolution accuracy of the impedance, we need to screen the interdigital electrode with different interdigital spacing to obtain the optimal invasion cell impedance spectrum Z_cells_. Figure 3a–c shows cell-free and cell-electrode impedance spectra from 100 Hz to 1000 kHz at 5 μm, 30 μm and 50 μm interfinger spacing interfinger electrodes, respectively. Impedance measurements of the same sample were performed using interfinger electrodes with 5 μm, 30 μm, and 50 μm interfinger spacing, respectively. Out of the 5 μm, 30 μm, 50 μm interdigital electrodes, the 5 μm interdigital electrode is not sensitive to cells, but the 30 μm interdigital electrode has better cell impedance resolution accuracy compared with the 30 μm and 50 μm electrodes. This may be due to the size of the measured Hela cells, which are usually around 20 μm in diameter [26], and the 5 μm electrical and extreme spacing is far smaller than the cell size and cannot characterize the overall characteristics of the cell. Although the 50 μm electrode meets the size requirements, its spacing is too large, and the measurement area contains more cell and medium contributions, resulting in the signal being averaged, resulting in a low impedance resolution of its measurement, so 30 μm is a moderate choice.

To find out which frequency has the most stable output and is most sensitive to cells, we calculated the impedance spectra corresponding to Z_no-cell_, Z_cell-electronic_, Z_cells_/Z_no-cell_ when selecting a 30 μm interdigital electrode. As shown in Figure 3d, the first two impedances decrease with an increase in frequency. The impedance of the present cells was higher than that of the non-present cells. This higher impedance is mainly because cancer cells adhere to the bottom surface of the chamber after invasion and are detected by electrodes. Z_cells_/Z_no-cell_ increases first and then decreases in the range of 100 Hz–100 kHz. When f_0_ = 1 kHz, the impedance curve of Z_cells_/Z_no-cell_ reaches the highest, and the impedance detection is also in a relatively stable state. It shows that the impedance of cells at 1 kHz has the highest resolution compared to that of cells without cells.

In the previous calculation, we screened three different specifications of electrodes and obtained the frequencies that the cells were most sensitive to. The experiment shows that the 30 μm electrode has good cell impedance resolution accuracy at 1 kHz. Therefore, in the following experiments under various conditions, we chose 1 kHz as the measurement frequency.

Since the measurement needs to be continuously monitored for a long time, to eliminate the impedance drift caused by external factors, we continuously monitor the impedance value of the blank chip at different flow rates at 1 kHz. As shown in Figure 3e, in a static state with a flow rate of 0, the impedance first continues to rise and then remains stable, while the impedance applying a steady flow rate also remains stable. This is because in the static state where the flow rate is 0, the ion migration rate near the electrode is greatly reduced, and the ion depletion layer or enrichment layer is easily formed at the electrode interface, resulting in a significant increase in the electrode polarization impedance [27,28]. At the same time, the electrode surface is more likely to adsorb pollutants in the solution or generate reaction products, such as oxide layer and biofilm. This pollution increases the interface impedance, causing the overall impedance to rise. The double electric layer on the electrode surface also gradually stabilizes and thickens, showing that the interface capacitance increases and affects the total impedance. The convection of fluid will break the concentration gradient and thin the diffusion layer. Increasing the flow rate can improve the ion exchange conditions on the electrode surface so that the thickness of the double electric layer is reduced or restored to a more stable state, to maintain the impedance stability. Since the final impedance remains stable in the static state and the subsequent detection is mostly in the fluid state, the drift of electrode impedance will not affect the subsequent modeling and verification process.

### 3.3. Cancer Cell Invasion Electrochemical Sensor-Counting Model

**Figure 4:**
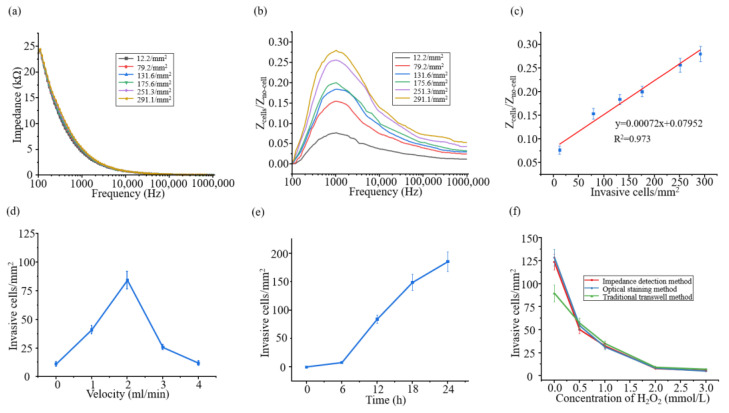
(**a**) Z_cell-electronic_ impedance spectrum corresponding to different invasion capabilities. (**b**) Z_cells_/Z_no-cell_ spectrum from 100 Hz to 1000 kHz corresponding to different invasion capabilities. (**c**) The corresponding relationship between Z_cells_/Z_no-cell_ and the invasion ability of cancer cells at *f*_0_ = 1 kHz; (**d**) the changes in the invasion ability of cancer cells at different flow rates for 12 h; (**e**) the sequential changes in the invasion ability of cancer cells at 2 mL/min flow rates. (**f**) The effects of different concentrations of hydrogen peroxide on the invasion ability of Hela cells were evaluated by electrical impedance detection, optical staining, and the Transwell method.

In this paper, to establish the relationship between the invasion number of different cancer cells and the corresponding impedance, we used this system to detect the cell impedance of different invasion numbers of cancer cells. Figure 4a shows the Z_cell-electronic_ impedance spectrum of different cancer cell invasion capabilities at 100 Hz–100 kHz. It can be seen from the figure that the more cancer cells invade, the higher the impedance. However, the impedance resolution accuracy of Z_cell-electronic_ is not high. Therefore, Z_cells_/Z_no-cell_ were used to distinguish the invasion ability of different cancer cells. It can be clearly seen from Figure 4b that the higher the Z_cells_/Z_no-cell_ detected, the more cancer cells invaded, and Z_cells_/Z_no-cell_ reached a peak value at 1 kHz. In Figure 4c, when *f*_0_ = 1 kHz, the corresponding relationship between Z_cells_/Z_no-cell_ and cancer cell invasion ability was established, and the fitting function was y = 0.00072x + 0.07952, where R^2^ was 0.973, showing a good correlation.

**Figure A1:**
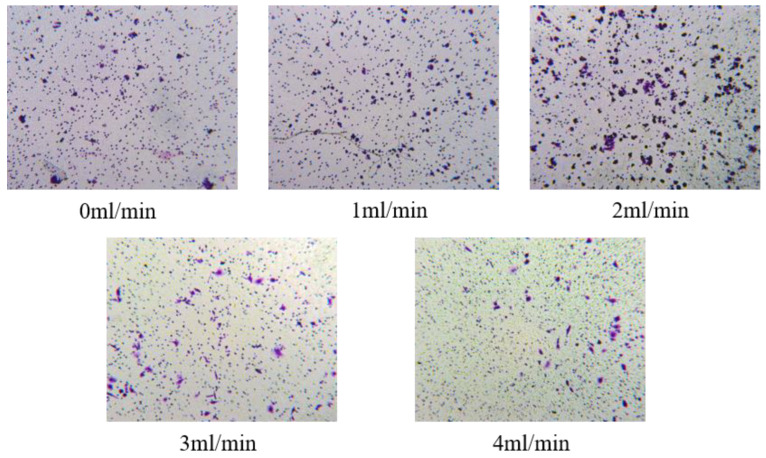
Micrograph of changes in the invasive ability of cancer cells treated with different flow rates for 12 h.

**Figure A2:**
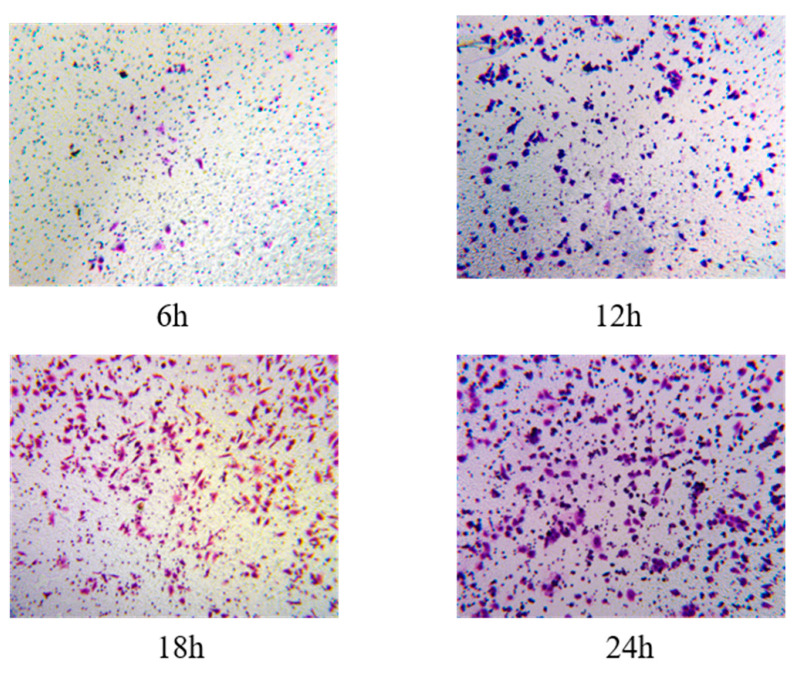
Micrograph of continuous changes in cancer cell invasiveness at a flow rate of 2 mL/min.

We detected the changes in cancer cell invasion ability at different flow rates for 12 h. As shown in Figure 4d and Figure A1, the number of cancer cell invasions first increased, reached the maximum at 2 mL/min, and then showed a downward trend. This is because at low flow velocity, fluid flows through cells to generate fluid shear stress, and mechanical stress stimulates cells, which can enhance the invasion ability of cancer cells [29,30,31,32]. However, cells adhere to the bottom of the cell, and at high flow rate, the shear stress generated by the fluid makes the cancer cells unable to maintain the state of adhesion and then escape, which shows that the invasion ability of cancer cells decreases, which is not inconsistent with the enhancement of the invasion ability of cancer cells by the fluid shear stress. Since the invasion quantity of Hela at the flow rate of 2 mL/min had the largest resolution compared with that at the static flow, we monitored the temporal changes in cancer cell invasion ability at the flow rate of 2 mL/min. As shown in Figure 4e and Figure A2, the invasion quantity of cancer cells increased with time, increasing significantly after 6 h and slowing down at 24 h. Based on the screening of the above two experimental conditions, we designed an impedance detection validation experiment based on cytotoxicity.

**Figure A3:**
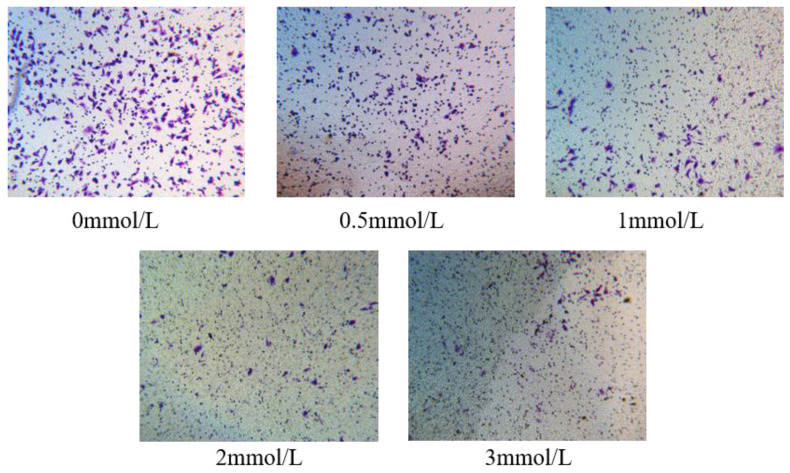
Optical staining micrograph of the effect of different concentrations of hydrogen peroxide on the invasive ability of Hela cells evaluated by the impedance method.

**Figure A4:**
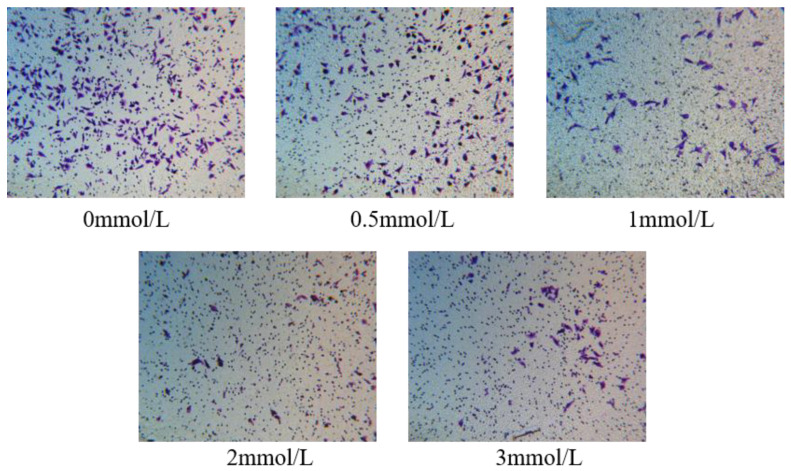
Micrograph of the effect of different concentrations of hydrogen peroxide on the invasive ability of Hela cells evaluated by the Transwell method.

**Figure A5:**
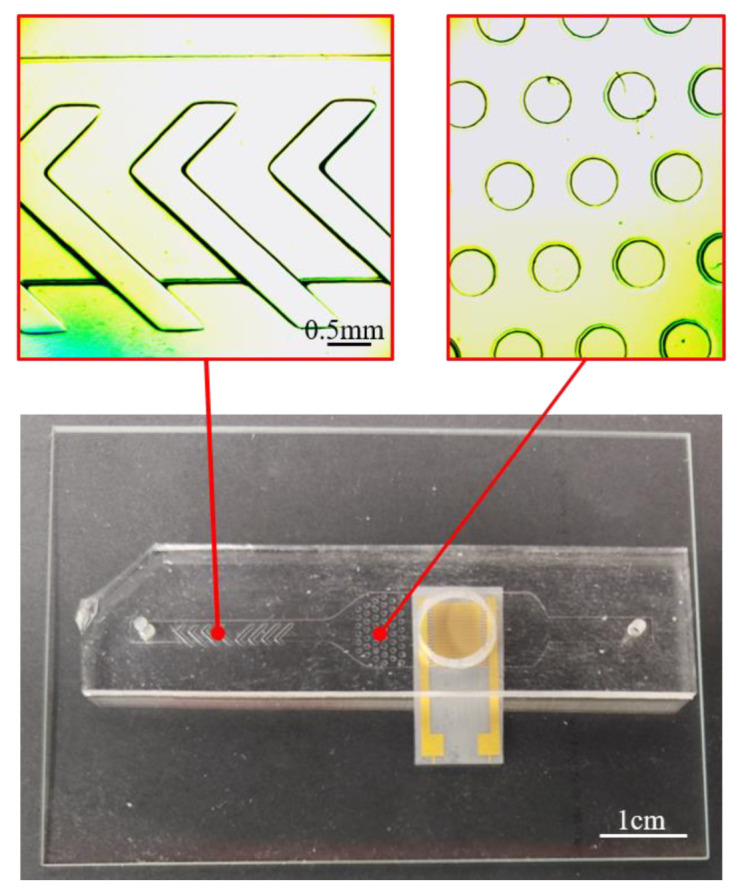
Physical picture of microfluidic chip and channel micrograph.

To verify the effectiveness and accuracy of this model, we used electrical impedance detection, optical staining, and the traditional Transwell method to evaluate the influence of different concentrations of hydrogen peroxide on the invasion ability of Hela cells. Electrical impedance detection and optical staining were selected to invade 2 mL/min for 24 h, while the traditional Transwell method was static for 24 h. As shown in Figure 4f, Figure A3 and Figure A4, the invasion ability of Hela cells detected by electrical impedance detection, optical staining, and the traditional Transwell method all decreased with the increase of hydrogen peroxide concentration, indicating that hydrogen peroxide can significantly affect the invasion ability of Hela cells. Among them, the electrical impedance detection method and the optical staining method are dynamic environment detection processes, and the number of invasions is higher than that of the traditional Transwell method when no drug is added, but it becomes lower than that of the traditional Transwell method after drug addition, which is due to the uniform fluid mixing in dynamic environment, which leads to the intensification of drug diffusion and reaction. The results showed that fluid could enhance the blocking effect of hydrogen peroxide on cancer cells, while the increase in drug concentration weakened the induction of the flow rate on cancer cell invasion. The optical staining method is our validation of the electrical impedance detection method, and their Pearson correlation coefficient is 0.9994, indicating a strong correlation between the two. Meanwhile, we calculate the mean square error, root mean square error, and range-based relative error, which are 69.33, 8.33, and 1.89%, respectively, indicating that the error between the two methods can be considered very low. Therefore, we believe that the established model of cancer cell invasion ability evaluation based on electrical impedance is effective and accurate.

### 3.4. Superiority Analysis

To investigate the advantages and limitations of previous methods for assessing cancer cell invasiveness, electrochemical impedance methods, and proposed methods, several important metrics were selected, such as incubation time, detection time, accuracy, invasiveness, and cost. Table 1 shows the results of the comparison. For traditional Transwell methods and fluorescent labeling methods, invasion is a common feature, which may cause cell damage to a certain extent. At the same time, tedious manual operation is required, resulting in a long detection time. In addition, the cultivation time and cost requirements of fluorescent staining are higher. Electrochemical impedance methods are label-free and non-invasive, with high accuracy for current cell assays. However, they still rely on static data collection and ignore the feedback of environmental factors on cell dynamic behavior. Meanwhile, due to the limitations of space and electric field distribution, they require custom electrodes, which also makes their cost much higher than our self-assembly method. Based on the existing impedance methods, our method establishes an electrical impedance model of cancer cell invasion, eliminates environmental interference, further reduces detection time, maintains high accuracy and moderate cost, and is suitable for the detection of cancer cell invasion. Table 1 shows that the proposed method has better performance, while the other methods have one or more drawbacks.

## 4. Conclusions

This paper proposes an electrical impedance-based evaluation method for assessing cancer cell invasion ability. A simple integrated interdigital electrode, microfluidic chip, and Transwell chamber are employed to rapidly and effectively assess cancer cell invasion ability in a dynamic fluid environment. We simulated and optimized the microfluidic chip structure to facilitate cancer cell invasion and impedance detection and identified the 30 μm interdigitated electrode with high impedance resolution as optimal for HeLa cells through comparison. To achieve optimal impedance differentiation, we determined that the impedance-sensitive frequency for HeLa cells is 1 kHz. At this frequency, we established a relationship between Z_cells_/Z_no-cell_ and cancer cell invasion ability, with the fitting function y = 0.00072x + 0.07952 and an R^2^ value of 0.973, indicating a strong correlation. Using this model, we conducted a preliminary drug screening application by employing hydrogen peroxide to establish a concentration gradient for cytotoxicity experiments. Optical staining was used to verify the results of the electrical impedance detection method. The Pearson correlation coefficient, mean square error, root mean square error, and range-based relative error were 0.9994, 69.33, 8.33, and 1.89%, respectively, indicating a high correlation between the two methods and very low error. The experimental results validate the high reliability of this model, and the system offers advantages such as short detection time, low cost, non-invasiveness, and lack of manual operation. Although our evaluation method is more efficient than conventional methods, it is still necessary to further optimize the durability of the electrode material and ensure its stability in long-term experiments. Optimizing the design of microfluidic chips to enable high-throughput processing for large-scale drug screening and multi-sample processing is also the focus of our further improvement. We also hope to introduce more environmental factors to simulate the biomimetic environment and comprehensively evaluate the invasion ability of cancer cells.

## Figures and Tables

**Table 1:** Comparison with other methods.

Method	Specific Application	Detection Object	Incubation Time	Detection Time	Accuracy	Intrusion or Not	Cost
Transwell method	Traditional Transwell model	Cell invasion	24 h	>30 min	High	√	Low
Fluorescent staining	Co-culture model [9]	Cell invasion	6 day	>6 h	High	√	High
	Tumor-vascular model [11]	Cell endocytosis	6 day	>8 h	High	√	High
Electrochemical impedance method	Static cell adhesion model [16]	Cytotoxicity	24 h	40 s	High	×	High
	Electric cell–substrate impedance sensing model [17]	Cell viability and vitality	24 h	20 min	High	×	High
The method proposed in this paper	Microfluidic Transwell model	Cell invasion	24 h	<10 s	High	×	Medium

## Data Availability

The data presented in this study are available on request from the corresponding author.
